# The Gemini Eye in Microsurgery: Video-Based Capillary Refill and Chromatic Assessment for Free Flap Monitoring

**DOI:** 10.3390/jcm15156114

**Published:** 2026-08-06

**Authors:** Ebru Aşiret, Burak Yaşar, Büşra Taş Efe, Süleyman Ege Tozan, Hasan Murat Ergani, Ramazan Erkin Ünlü

**Affiliations:** Department of Plastic, Reconstructive and Aesthetic Surgery, Ankara Bilkent City Hospital, Ankara 06800, Turkey; burakys@outlook.com (B.Y.); tasbusrabusratas@gmail.com (B.T.E.); egetozan@gmail.com (S.E.T.); dr.hasanmrt_06@hotmail.com (H.M.E.); rerkinunlu@yahoo.com (R.E.Ü.)

**Keywords:** free flap monitoring, artificial intelligence, capillary refill time, structured prompting, postoperative surveillance, vascular compromise, large language model, adjunctive monitoring

## Abstract

**Background/Objectives**: Postoperative free flap monitoring relies on clinical assessment of colour, turgor, and capillary refill time (CRT). The high frequency of required assessments renders this process labour-intensive and inherently subjective, with sensitivity dependent on observer experience and fatigue. This study evaluated the feasibility of a large multimodal AI (Artificial Intelligence) model (Gemini 3 Flash, Google AI Studio) applied without any task-specific training or fine-tuning, with each video analysed in an independent session, to establish whether meaningful diagnostic agreement is achievable before any domain-specific training is introduced, with the ultimate goal of supporting the development of a machine-based secondary safety net that augments, rather than replaces, the primary clinical assessment of the responsible surgeon in postoperative free flap surveillance. **Methods**: One hundred and forty-three postoperative video recordings from 143 different patients who underwent fasciocutaneous free flap reconstruction for extraoral defects were analysed. The cohort was deliberately enriched for pathological cases to ensure adequate representation of each vascular compromise category. Assessment was based on intrapatient comparison between flap and adjacent native tissue, without task-specific training or prior clinical information. AI outputs were compared against consensus assessments of three senior plastic surgeons (two associate professors, one full professor) for four parameters: flap colour, turgor, CRT interpretation, and clinical diagnosis. Agreement was evaluated using Cohen’s kappa (κ). Receiver operating characteristic (ROC) analysis assessed the discriminative performance of the AI confidence score. **Results**: Statistically significant agreement was demonstrated across all four parameters (*p* < 0.001 for all): flap colour (κ = 0.613, 95% CI: 0.489–0.737, 80.4%), turgor (κ = 0.676, 95% CI: 0.558–0.794, 83.9%; assessed from visual surrogates rather than direct palpation), CRT interpretation (κ = 0.487, 95% CI: 0.360–0.614, 74.1%), and clinical diagnosis (κ = 0.524, 95% CI: 0.399–0.649, 74.1%). Normal flaps were correctly identified in 81.0% of cases and venous compromise in 69.4%. ROC analysis identified a confidence score percentile cutoff of 89 as the optimal threshold for discriminating compromised from normal flaps, with scores below 89 indicating a compromised (pathological) result (AUC = 0.667, 95% CI: 0.579–0.755; sensitivity 77.0%, specificity 52.4%; overall accuracy 62.9%). **Conclusions**: A structured-prompted multimodal LLM (Large Language Model) demonstrated statistically significant agreement with the consensus judgement of senior plastic surgeons in postoperative free flap assessment without task-specific training, relying solely on intrapatient visual comparison. These findings constitute a proof-of-concept for AI-based video analysis as a potential adjunctive approach in free flap surveillance, warranting prospective validation with independent clinical outcome data before any claim of clinical utility, with possible broader applicability across clinical domains pending such validation. Key limitations include the absence of independent clinical outcome or confirmation of diagnostic categories, deliberately enriched sampling, a small arterial insufficiency subgroup, and the current absence of a formal data processing agreement for the cloud-based AI platform used.

## 1. Introduction

Free tissue transfers are widely used for aesthetic and functional reconstruction of soft tissue, composite, and bony defects, with indications spanning burn injury, trauma, oncological resection, and congenital anomalies.

Flap viability depends entirely on the integrity of the microvascular anastomoses during the first 7–10 postoperative days, before neovascularisation is established [1,2]. Arterial and venous compromise is the leading cause of free flap loss and typically develops within the first 48 postoperative hours. Venous compromise is more frequently encountered than arterial insufficiency and constitutes the most common precipitant of flap los [2,3,4,5,6] Early recognition of vascular compromise is essential, as prompt intervention prevents flap loss. Postoperative monitoring is therefore a critical component of free flap management [7].

Postoperative monitoring is most commonly performed through clinical assessment of colour, turgor, temperature, capillary refill time (CRT), and acoustic Doppler signal [2,8]. Clinical assessment remains the gold standard of flap surveillance [8]; however, current protocols mandate assessment every one to two hours during the first 48 postoperative hours—a surveillance burden that, in high-volume units, falls disproportionately on junior residents during overnight periods, under conditions of fatigue and without immediate senior oversight [4,5,7]. Alternative monitoring modalities, including infrared thermography, have been explored but have not achieved widespread clinical adoption [9].

Given the critical importance of timely flap surveillance and the demonstrated impact of early intervention on flap salvage, reducing dependence on observer experience and inter-rater variability constitutes an important clinical objective. An adjunctive automated monitoring system may address this need, and artificial intelligence offers a promising approach toward this goal.

Large multimodal models may be capable of approximating certain human perceptual tasks and are not subject to fatigue-related decline in performance. However, they remain susceptible to other forms of bias, including those inherited from training data and algorithmic design, and their reliability across diverse clinical contexts remains to be established [10,11,12,13]. AI-based video analysis systems may therefore enable the development of adjunctive monitoring tools that support primary clinical assessment in postoperative free flap surveillance.

Motivated by these considerations, we investigated whether a general-purpose multimodal AI model could assess postoperative free flap videos in a clinically meaningful manner without any task-specific training or fine-tuning. Gemini 3 Flash (Google AI Studio) was selected for this purpose, as it supported native video upload and independent session-based analysis. By establishing the baseline performance of an untrained model, we aimed to demonstrate what may be achievable when such a model is further trained or refined for this specific clinical task.

## 2. Materials and Methods

### 2.1. Study Design and Ethics

This retrospective observational study was conducted at our tertiary referral center for plastic surgery under the Ankara Bilkent City Hospital Medical Research Scientific and Ethical Evaluation Board approval (TABED 2/1945/2026, date: 4 March 2026), which explicitly covered the upload and processing of anonymised video recordings through a third-party, cloud-based AI platform (Google AI Studio). Individual informed consent was waived as the study involved retrospective analysis of anonymised video recordings obtained during routine clinical care. All recordings were fully anonymised (with no visible facial features, tattoos, identification bands, or other patient-identifying elements) prior to upload and analysis, consistent with local data protection requirements. Per Google AI Studio’s stated data-handling terms at the time of access, uploaded content was not retained by the platform beyond the analysis session. No separate data processing agreement was in place between the institution and Google. This absence of a formal agreement is acknowledged as a limitation of the current data governance framework (see Limitations). This study was conducted in accordance with the Declaration of Helsinki.

### 2.2. Assessment Parameters

Each video was evaluated for four parameters (flap colour, turgor, CRT interpretation, and clinical diagnosis) by both the clinical panel and the AI model. Classification criteria and their clinical correlations are presented in Table 1.

### 2.3. AI-Based Assessment

Each video was analysed using Gemini 3 Flash (version: gemini-3-flash-001; Google LLC, Mountain View, CA, USA), accessed via the Google AI Studio web interface, with videos manually uploaded and analysed in April 2026. Gemini was selected for its native full-video upload capability and continuous temporal analysis, which are essential for physiologically valid CRT quantification. Model temperature was set to 0.1 to minimise output variability. A structured, stepwise prompt (Table 2) was applied uniformly across all videos: the video was first uploaded to the Google AI Studio chat interface, and the full prompt text was then pasted into the chat input as a single message immediately following the video, with the model returning its complete structured response in a single turn (i.e., not as a multi-turn, step-by-step exchange). No clinical information was provided; all assessments were based solely on visual analysis. In addition to categorical assessments, the AI model was prompted to provide a confidence score (0–100) for each evaluation, reflecting its own certainty in the assigned classification. This was incorporated to determine whether the model could not only classify flap status but also indicate how reliably it was able to do so—effectively providing a self-reported measure of output quality for each individual case. The AI was instructed to output colour estimates at RGB/hex resolution and CRT at millisecond resolution (Table 2) to support fine-grained intrapatient comparison; RGB/hex values were used qualitatively to compare flap chromaticity against adjacent healthy skin within the same frame, and millisecond-level CRT values were converted to seconds and mapped to the categorical bins used in the primary analysis (Table 1)—for example, a computed value of 3.4 s was assigned to the “prolonged” category. The raw RGB/hex and millisecond-level outputs were not themselves subjected to formal statistical analysis in the present study but were retained as a supplementary dataset for future quantitative calibration work.

**Figure 1:**
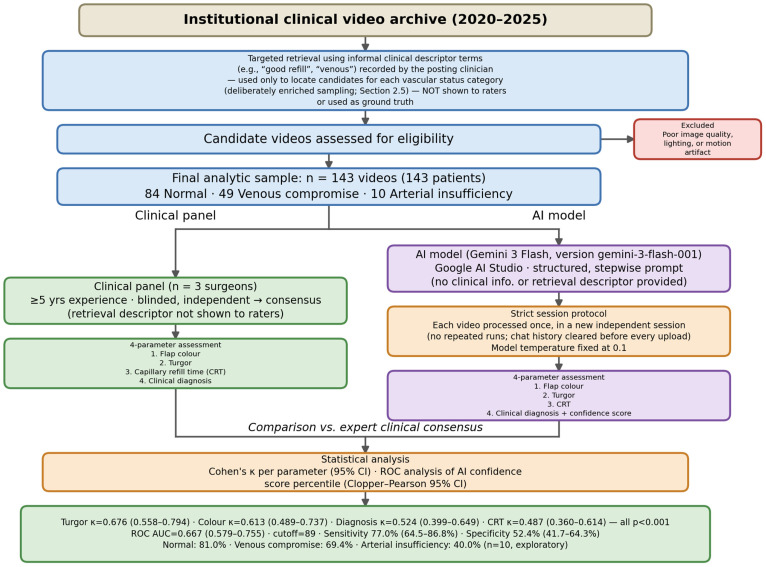
Study flowchart. A total of 143 postoperative free flap video recordings were retrospectively retrieved from the institutional clinical video archive (2020–2025) using clinical descriptor search terms corresponding to each vascular status category, consistent with the deliberately enriched sampling design (Section 2.5). These search terms reflected an informal clinical impression recorded by the posting clinician at the time of upload (e.g., “good refill”, “venous”) and were used only to locate candidate videos; they were not shown to either rater and were not used as ground truth, since some such impressions—particularly those recorded by more junior team members—were later found not to match the eventual clinical course. Videos were excluded if image quality, lighting conditions, or motion artifacts precluded reliable assessment. The remaining recordings were evaluated in parallel by two independent raters, both blinded to the retrieval descriptor: (1) a clinical panel of three plastic surgeons with a minimum of five years of microsurgical free flap experience, each reviewing all videos independently and blinded to one another’s assessments, with disagreements resolved by consensus, and (2) a large multimodal AI model (Gemini 3 Flash, Google AI Studio) applying a structured, stepwise prompt uniformly across all videos. To prevent contextual carry-over between assessments, each video was processed in a new, independent session with the chat history cleared prior to every upload. Model temperature was fixed at 0.1 to minimize output variability. No clinical information was provided to the model; all assessments were based solely on visual analysis. Both raters evaluated four parameters: flap colour, turgor, capillary refill time (CRT), and overall clinical diagnosis. AI outputs were additionally assigned a numerical confidence score (0–100). Agreement between AI and clinical consensus was quantified using Cohen’s kappa (κ) for each parameter, and receiver operating characteristic (ROC) analysis was performed to determine the optimal confidence score threshold for discriminating compromised from normal flaps. CRT: capillary refill time; AUC: area under the curve; κ: Cohen’s kappa coefficient.

Assessment was based on intrapatient comparison between flap tissue and adjacent native skin visible within the same video frame, without any prior knowledge of the patient’s baseline skin tone, flap type, or clinical history. The model received no task-specific training and no contextual information beyond the video itself, reflecting the baseline performance of a general-purpose AI system applied without any domain-specific preparation. Each video was processed once, in a single independent session, ensuring that the model had no access to previously analysed cases and could not learn from prior assessments (Figure 1); no repeated runs were performed per video, so run-to-run reproducibility of individual outputs was not formally assessed in this study (see Limitations). The AI returned a structured free-text response following the detailed verbatim prompt in Table 2 (also provided as Supplementary File S1 in downloadable form), from which categorical classifications, the AI-calculated CRT value, and the confidence score were manually extracted into the study dataset. Free-text colour descriptors returned by the model (e.g., Pale, Dusky, Cyanotic, Blue/Purple, as shown in the verbatim prompt in Table 2) were mapped by the study team onto the three formal colour categories defined in Table 1 (Pale/White, Pink, Blue/Purple) for quantitative analysis. Videos for which the model produced a failed, incomplete, or ambiguous output that could not be mapped to the required categories were excluded, together with videos excluded during the archive-based selection process described in Section 2.5 for image quality, lighting, or motion artifact.

### 2.4. Clinical Assessment

All 143 videos were independently reviewed by three senior plastic surgeons—comprising two associate professors and one full professor of plastic and reconstructive surgery—each with a minimum of five years of dedicated microsurgical free flap experience, blinded to AI outputs and to each other’s assessments. In cases of interrater disagreement, a consensus decision reached through discussion served as the expert clinical consensus; this consensus was not independently confirmed against objective clinical outcomes such as intraoperative findings, Doppler assessment, or flap salvage/loss, and this study should accordingly be interpreted as a pilot agreement study rather than a validated diagnostic performance study. Turgor was assessed visually from video recordings based on observable signs including rate of skin return following instrument compression, presence of tissue oedema, skin tightness, and blister formation—parameters identifiable from video without direct tactile examination.

### 2.5. Video Archive and Patient Selection

Postoperative free flap monitoring videos, routinely recorded by the plastic surgery team as part of standard clinical surveillance, are backed up monthly to an institutional clinical hard-drive archive. For this retrospective study, candidate videos were retrieved from this archive using clinical descriptor search terms corresponding to each vascular status category (normal, venous compromise, arterial insufficiency), in order to achieve adequate representation of each category for subgroup analysis; this targeted retrieval process is consistent with the deliberately enriched sampling design described below. These search terms reflected an informal clinical impression recorded by the posting clinician at the time of upload and were used only to locate candidate videos; they were not shown to either rater and were not used as ground truth, as some such impressions—particularly those recorded by more junior team members—were later found not to match the eventual clinical course. A total of 143 video recordings were selected from this archive, comprising 143 different patients who underwent fasciocutaneous free flap reconstruction for extraoral defects between 2020 and 2025. Videos were excluded if image quality, lighting conditions, or motion artifacts precluded reliable assessment, or if patient-identifying features were visible in the frame. Videos in which the adjacent native tissue was not clearly visible for intrapatient comparison were also excluded. Videos recorded using a smartphone under natural daylight conditions, with a minimum duration of 10 s, at a distance of 25 ± 5 cm perpendicular to the flap surface, and in which a surgical instrument compression maneuver was performed and released during recording to enable CRT assessment, were selected. Full video acquisition parameters are summarised in Table 3.

The AI assessment methodology relied on intrapatient comparison—evaluating the flap against the adjacent native skin visible within the same video frame, rather than on absolute colour values. We assumed that variations in device type or lighting conditions would affect both the flap and the native skin comparably, and therefore would not introduce a systematic bias into the intrapatient comparison; however, this assumption was not empirically tested in the present study, and device/illumination standardization was not enforced beyond the acquisition parameters in Table 3. Future work should formally test the robustness of AI-based intrapatient comparison across device types and lighting conditions.

All included flaps were fasciocutaneous in type; musculocutaneous, osteocutaneous, chimeric, and conjoined flaps were not represented in this cohort. To ensure adequate statistical power for subgroup analysis of vascular compromise categories, the cohort was deliberately enriched for pathological cases, as informed by a priori power calculations. The distribution of normal, venous compromise, and arterial insufficiency cases therefore reflects the study sampling design and does not represent the institutional complication rate.

### 2.6. Statistical Analysis

Agreement between AI outputs and the clinical consensus was evaluated using Cohen’s κ, interpreted as: <0.20 slight, 0.21–0.40 fair, 0.41–0.60 moderate, 0.61–0.80 substantial, and >0.80 near-perfect agreement. IBM SPSS Statistics reports Cohen’s κ and its asymptotic standard error but does not provide the corresponding 95% confidence interval; therefore, confidence intervals for κ were calculated from the asymptotic standard error using the normal approximation (κ ± 1.96 × SE). ROC analysis assessed the AI confidence score (a self-reported numerical estimate, 0–100, indicating the model’s certainty in its own assessment) in discriminating compromised from normal flaps, with confidence score values below the identified cutoff taken as the diagnostic output for a pathological (compromised) result. The confidence score reflects how certain the model was in its own interpretation of the video—that is, how confidently it assessed the case—regardless of which category it ultimately assigned. A high score therefore does not indicate that the AI judged the flap to be normal or compromised specifically; it indicates only that the AI was confident in whatever classification it gave. Empirically, lower confidence scores were more often associated with the AI’s own classification of a flap as compromised; this raw score was therefore used directly in the ROC analysis, with lower values taken as more consistent with a pathological classification. Consequently, this ROC analysis is best interpreted as an exploratory association between expressed model certainty and classification outcome, with a role closer to an error/calibration signal than an independent diagnostic discriminator. 95% confidence intervals for sensitivity, specificity, PPV, and NPV were estimated using the Clopper–Pearson exact method; F1-scores are reported as point estimates only, as this composite metric does not admit a direct Clopper–Pearson interval. Descriptive statistics are reported as mean ± SD and median with range. All analyses, including the ROC, Clopper–Pearson exact confidence intervals, and asymptotic confidence intervals for κ, were performed using SPSS version 26.0 (IBM Corp., Armonk, NY, USA). This study’s design and reporting were informed by the CLAIM (Checklist for Artificial Intelligence in Medical Imaging) framework [14].

## 3. Results

A total of 143 postoperative free flap video recordings were analysed: 84 normal flaps (58.7%), 49 venous compromise (34.3%), and 10 arterial insufficiency (7.0%).

### 3.1. Flap Colour Assessment

Statistically significant agreement was demonstrated for flap colour (κ = 0.613, 95% CI: 0.489–0.737, 80.4%, *p* < 0.001) (Table 4). Among 96 pink flaps, 78 (81.3%) were correctly identified; of 43 blue/purple flaps, 33 (75.0%) were accurately classified. Pale/White flaps demonstrated the lowest concordance (4 of 14 cases; 28.6%).

### 3.2. Turgor Assessment

Substantial agreement was observed for turgor (κ = 0.676, 95% CI: 0.558–0.794, 83.9%, *p* < 0.001) (Table 5). The AI correctly identified 47 of 54 high-turgor flaps (87.0%) and 73 of 80 normal-turgor flaps (91.3%). As turgor was assessed from visual surrogates rather than direct palpation in both the AI and the clinical panel, this agreement reflects concordance in visually inferred turgor rather than validated tactile assessment.

### 3.3. Capillary Refill Time

Moderate agreement was demonstrated for CRT interpretation (κ = 0.487, 95% CI: 0.360–0.614, 74.1%, *p* < 0.001) (Table 6). Of 86 flaps with clinically normal CRT, 72 (83.7%) were correctly categorised. The AI showed a consistent tendency toward over-classification of prolonged CRT: only 5 of 19 AI-designated prolonged cases (26.3%) were clinically confirmed, while 10 (52.6%) were in fact normal. Mean AI-calculated CRT was 1.59 ± 1.25 s (median 1.4 s, range 0.3–9.4 s). This AI-calculated CRT value in seconds was not itself analysed as a continuous outcome; rather, it served as the basis for deriving the categorical CRT classification (Prolonged, Normal, or Brisk; Table 1) used in the primary agreement analysis. Mean confidence score was 87.54 ± 5.42 (median 88, range 55–95) (Table 7).

### 3.4. Clinical Diagnosis and Diagnostic Agreement

**Figure 2:**
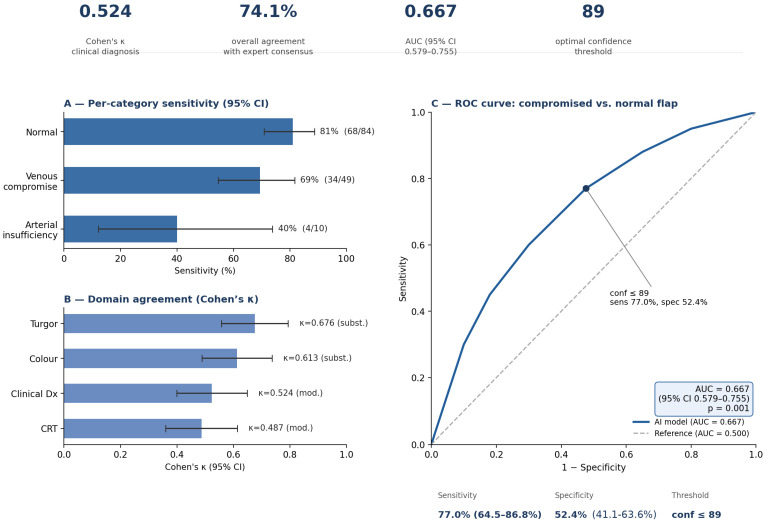
Composite summary of AI agreement with expert clinical consensus for free flap perfusion assessment across 143 video recordings. (**A**) Per-category sensitivity (detection rate): the AI correctly identified 81% of normal flaps, 69% of venous compromise cases, and 40% of arterial insufficiency cases; the latter should be interpreted with caution given the small cohort (n = 10). (**B**) Cohen’s kappa (κ) by assessment domain: turgor (κ = 0.676) and colour (κ = 0.613) achieved substantial agreement; clinical diagnosis (κ = 0.524) and capillary refill time (κ = 0.487) reached moderate agreement. (**C**) ROC curve for discrimination of compromised versus normal flaps. AUC = 0.667 (95% CI: 0.579–0.755; *p* = 0.001). A confidence score threshold of 89 was identified as the optimal operating point (sensitivity 77.0%, 95% CI: 64.5–86.8%; specificity 52.4%, 95% CI: 41.1–63.6%) using the Youden J statistic. The dashed line represents the reference classifier (AUC = 0.500). Dx, diagnosis; CRT, capillary refill time; AUC, area under the receiver operating characteristic curve; conf, AI confidence score; subst., substantial agreement; mod., moderate agreement.

Moderate agreement was observed for clinical diagnosis (κ = 0.524, 95% CI: 0.399–0.649, 74.1%, *p* < 0.001) (Table 8). The AI correctly identified 68 of 84 normal flaps (81.0%), 34 of 49 venous compromise cases (69.4%), and 4 of 10 arterial insufficiency cases (40.0%). Per-class sensitivity, specificity, positive and negative predictive values, and F1-scores for this categorical diagnosis are presented in Table 9, with arterial insufficiency estimates (n = 10) interpreted as exploratory given their wide confidence intervals; a binary analysis of compromised versus normal flaps based on this categorical diagnosis is presented in Table 10 (Figure 2A,B).

### 3.5. Confidence Score-Based Classification

As a separate analysis, ROC analysis was constructed with a pathological (compromised) result as the target outcome and identified a confidence score cutoff of 89 as the optimal threshold, with scores below 89 taken as diagnostic for a pathological result and scores above 89 as diagnostic for a normal result (AUC = 0.667, 95% CI: 0.579–0.755; *p* = 0.001) (Figure 2C). Full classification metrics for this confidence-score-based classifier are presented in Table 11: for the pathological target class, sensitivity was 77.0% (95% CI: 64.5–86.8%), specificity 52.4% (95% CI: 41.1–63.6%), PPV 54.7% (95% CI: 43.5–65.4%), NPV 75.4% (95% CI: 62.2–85.8%), and F1-score 0.644, with an overall accuracy of 62.9% (95% CI: 54.5–70.9%).

## 4. Discussion

This pilot study demonstrates the feasibility of a general-purpose multimodal LLM as a potential automated early-warning adjunct to conventional postoperative free flap surveillance. The model characterised free flap perfusion from unedited clinical recordings—without task-specific training, without prior patient information, and relying solely on intrapatient comparison between flap and adjacent native tissue—achieving moderate-to-substantial agreement with a senior clinician-established expert clinical consensus across all four assessed parameters (κ range: 0.487–0.676, *p* < 0.001 for all), correctly identifying 81.0% of normal flaps and 69.4% of venous compromise cases. To our knowledge, this is the first application of a structured-prompted LLM to video-based free flap monitoring. Existing AI studies in this domain have predominantly relied on still images [3,4,5]. Video recording captures the dynamic process of capillary refill (the temporal sequence of compression, blanching, and colour restoration) which is inherently time-dependent and cannot be reliably quantified from a static image. This marks a methodological advance over photograph-based paradigms.

This study does not propose AI as a replacement for clinical examination, which remains the gold standard of free flap surveillance and the sole basis for operative decision-making. Rather, it addresses a specific and underappreciated vulnerability in current monitoring protocols: the interval between scheduled assessments, particularly during overnight periods when surveillance responsibility rests with the most junior members of the clinical team. An automated system capable of continuous or high-frequency video-based screening and of alerting the responsible surgeon to potential vascular compromise—while remaining silent for normal flaps—would function as a secondary layer of safety without displacing clinical expertise. In this framework, the most operationally relevant performance metric is not overall diagnostic accuracy but specificity for the normal flap: the system must not wake the surgeon unnecessarily. The 81.0% correct identification rate for normal flaps observed in the present study, achieved without any model training, supports the viability of this concept.

Statistically significant agreement was demonstrated across all four parameters (κ range 0.487–0.676, *p* < 0.001 for all), indicating that a structured-prompted LLM can extract clinically relevant visual signal from free flap perfusion, warranting further prospective evaluation. The integration of AI into free flap monitoring has been explored through purpose-trained deep learning systems: Kim et al. achieved an AUC of 0.960 with DenseNet121 trained on 11,112 photographs [2], while machine learning models have also demonstrated utility in predicting flap failure and complications in breast and head and neck reconstruction [15,16] The application of large language models to surgical decision-making has similarly shown promise, including in the context of intraoperative queries and ICG fluorescence-guided tissue assessment [17,18]. Our study employed a generalist LLM applied to routine clinical videos without any task-specific training, mirroring real-world access conditions. Although the confidence-score-based ROC analysis in this cohort yielded a more modest AUC (0.667, 95% CI: 0.579–0.755) than purpose-trained convolutional models, the statistically significant kappa agreement observed consistently across all four assessment domains indicates that meaningful diagnostic signal can be extracted from an untrained, general-purpose model—a proof-of-concept finding that may improve substantially with task-specific fine-tuning.

The incremental value of this approach relative to existing monitoring methods lies less in raw diagnostic accuracy—where purpose-trained convolutional models such as that of Kim et al. [2] currently outperform a general-purpose, zero-shot LLM—and more in deployability. Purpose-trained models require large, institution-specific annotated datasets and dedicated machine-learning infrastructure to develop and maintain, resources that few plastic surgery units possess. Infrared thermography [9] and indocyanine green angiography [17,18] require dedicated hardware, consumables, and, for ICG, an invasive dye injection. By contrast, the present approach required no training data, no specialised hardware beyond a smartphone camera, and no in-house machine-learning expertise, and can in principle be redeployed to a new institution or task by modifying the prompt alone. This lower barrier to entry may make a general-purpose multimodal LLM a pragmatic starting point for centres lacking the resources to develop bespoke AI systems, even though its current classification accuracy remains inferior to purpose-trained alternatives.

These results were obtained without any domain-specific preparation. With task-specific training, fine-tuning, or dedicated application development, performance may be substantially improved. This suggests that a more reliable and clinically deployable secondary safety net for free flap surveillance may be achievable. Furthermore, the underlying approach—intrapatient, video-based visual comparison using a general-purpose foundation model—is not limited to free flap monitoring. It may be applicable to other clinical settings in which postoperative video-based assessment is routinely performed.

Venous compromise is the most frequent cause of postoperative flap failure [3,4,6]. The AI achieved a sensitivity of 69% for venous compromise, providing a clinically useful early-warning signal when embedded within an alert system designed to prompt (rather than replace) clinical assessment [2]. The statistically significant differentiation of AI-derived CRT across diagnostic groups (Kruskal–Wallis *p* = 0.0007) further supports this application: venous compromise exhibited the shortest median CRT (0.90 s; brisk refill) and arterial insufficiency the longest (4.05 s; prolonged), consistent with established clinical physiology.

The low prevalence of arterial insufficiency (n = 10; 7.0%) is epidemiologically representative—arterial complication rates of 2–4% are consistently reported in large series [3,6,19] However, the resulting F1-score of 0.33 precludes deployment as an autonomous arterial alert. Expansion of the arterial cohort to ≥30–50 cases is the single most critical priority for future work [12].

ROC analysis identified a confidence score cutoff of 89 (AUC = 0.667; sensitivity 77.0%, specificity 52.4%; overall accuracy 62.9%, 95% CI: 54.5–70.9%) (Table 11)—notably lower than the 79.0% accuracy achieved by the AI’s categorical diagnostic classification (Table 10), reinforcing that expressed confidence functions better as an error/calibration signal than as an independent diagnostic discriminator. This AUC reflects limited discriminative ability, and the accompanying specificity indicates that a substantial proportion of normal flaps would be flagged incorrectly if this threshold were used in isolation; a ceiling effect was also observed, as even incorrectly classified cases attracted a mean confidence of 84.9. However, cases with lower confidence scores were disproportionately associated with misclassification, suggesting an exploratory, hypothesis-generating association between expressed confidence and error rate rather than a validated calibration of the model’s output. This association may provide a potential basis for confidence-guided triage—for example, treating outputs below the cutoff of 89 as warranting mandatory clinical correlation—but this application has not been prospectively validated and should not be interpreted as demonstrated clinical utility. Future studies with formal calibration analyses and independent clinical outcome data would be required before such an approach could be considered for clinical implementation.

A fundamental constraint of the present design is that all 143 recordings were obtained from different patients, making longitudinal analysis of perfusion trajectory within a single patient impossible. Serial assessments from the same patient would have allowed identification of the earliest AI-detectable timepoint of deterioration and estimation of a clinically meaningful warning window—the interval between first signal change and the threshold for re-exploration [20]. The free flap salvage literature consistently identifies six hours of ischaemia as the critical boundary beyond which re-perfusion becomes unlikely, with venous compromise typically becoming clinically apparent within 30–90 min of onset [1,7]. An AI system capable of detecting compromise before gross clinical signs emerge could extend the actionable window, particularly during out-of-hours periods when observer experience is most variable.

Current monitoring protocols mandate clinical assessment every 1–2 h during the first 24–48 postoperative hours [3,4]. A video-capable AI monitoring system could, in principle, alter this framework by decoupling surveillance frequency from clinician availability. As a future direction that was not evaluated in the present dataset, an automated system could conceivably allow the responsible surgeon to define a higher-than-routine monitoring frequency—for example, checks every 15–30 min rather than hourly—for individual flaps judged to warrant closer observation, a level of surveillance intensity difficult to sustain with human staffing alone but technically achievable for an automated system, pending prospective evaluation of its clinical value and burden.

As a further, currently untested future direction, AI integration could enable risk-stratified monitoring frequency, an approach that purely human-based protocols cannot readily implement. Flaps at higher risk of vascular compromise (those with known technical difficulties at anastomosis, those in patients with significant comorbidities, or those demonstrating early borderline AI-derived parameters) could conceivably be assigned a higher monitoring frequency automatically, while stable low-risk flaps receive less intensive surveillance. This adaptive, risk-stratified concept mirrors the logic of continuous vital sign monitoring in intensive care, where alarm thresholds and sampling frequencies are individualised to patient acuity.

### Limitations

First, this was a single-centre retrospective study, and all included flaps were fasciocutaneous; musculocutaneous, osteocutaneous, chimeric, and conjoined flaps were not represented. Furthermore, Gemini 3 Flash is a proprietary commercial model whose underlying architecture and training data are not publicly disclosed, and whose performance characteristics may change with future vendor-driven updates outside the authors’ control. These factors may limit the generalisability of the present findings to other centres, flap types, and model versions.

Second, the cohort was deliberately enriched for pathological cases to ensure adequate representation of each vascular compromise category, and therefore does not reflect the institutional complication rate. Despite this enrichment, arterial insufficiency remained rare (n = 10, 7.0%), consistent with the low prevalence of arterial complications generally reported in the literature (2–4%) [3,6,19], underscoring the inherent difficulty of assembling a large arterial cohort even under enriched sampling. Additionally, all 143 recordings derive from different patients (cross-sectional design), preventing longitudinal trajectory modelling and re-exploration timing analysis within individual patients.

Third, videos were obtained under variable lighting, camera distances, and angles without a formal capture protocol; the performance metrics reported should therefore be interpreted as a lower bound on what standardised input might achieve.

Fourth, turgor assessment in the clinical setting is fundamentally a tactile parameter; direct palpation of the flap—the primary component of turgor evaluation—cannot be replicated from video. Both the clinical panel and the AI model relied exclusively on visual surrogates of turgor, including rate of skin return following instrument compression, tissue oedema, skin tightness, and blister formation. While these visual signs are identifiable from video and correlate with turgor status, the absence of palpation-based assessment reflects an inherent limitation of any video-only monitoring approach.

Fifth, the ground truth classification was assigned through clinical consensus without formal assessment of interrater reliability (IRR) among the evaluating clinicians, and diagnostic categories were not independently confirmed by objective clinical outcomes such as intraoperative findings at re-exploration, Doppler assessment, or flap salvage/loss; this consensus therefore constitutes a pilot agreement comparator rather than a validated clinical ground truth. Without IRR data, it is not possible to disentangle AI classification error from expert clinical consensus imprecision. Future prospective studies should incorporate a structured IRR protocol spanning junior to consultant level, report kappa values for each domain alongside AI agreement metrics, and, where possible, confirm diagnostic categories against independent clinical outcome data [21].

Sixth, the AI evaluated each flap as a homogeneous unit and was unable to selectively assess partially compromised zones demarcated by surgical marking, nor to exclude adjacent skin grafts from chromatic analysis—errors reflecting the absence of anatomical segmentation in a generalist model.

Seventh, Gemini outputs are probabilistic and non-deterministic; prompt rewording or model version updates may produce different results. Because each video was processed only once, run-to-run reproducibility of individual AI outputs was not formally assessed in the present study, and the reported findings cannot be assumed to generalise across repeated runs or model iterations.

Eighth, although Institutional Review Board approval explicitly covered the use of a third-party, cloud-based AI platform, and although uploaded videos were fully anonymised and were not retained by the platform beyond the analysis session per its stated terms, no formal data processing agreement was in place between the institution and Google at the time of this study. This reflects a gap in the current institutional data governance framework for cloud-based AI tools that should be addressed, through formal institutional-level data processing agreements, before this workflow is scaled beyond a retrospective research context.

## 5. Conclusions

A structured-prompted multimodal LLM demonstrated statistically significant agreement with the consensus judgement of senior plastic surgeons in postoperative free flap assessment without task-specific training, relying solely on intrapatient visual comparison. These findings constitute a proof-of-concept for AI-based video analysis as a potential adjunctive approach in free flap surveillance, warranting prospective validation with independent clinical outcome data before any claim of clinical utility, with possible broader applicability across clinical domains pending such validation.

## Figures and Tables

**Table 1:** Assessment parameters and classification categories.

Parameter	Categories	Clinical Correlation
Flap Colour	Pale/White	Arterial insufficiency
	Pink	Normal perfusion
	Blue/Purple	Venous compromise
Turgor/Oedema	Low	Arterial insufficiency
	Normal	Normal
	High	Venous compromise
Capillary Refill Time (CRT)	Prolonged (>2 s)	Arterial insufficiency
	Normal (1–2 s)	Normal
	Brisk (<1 s)	Venous compromise
Clinical Interpretation	Normal Arterial insufficiency Venous compromise	Expert clinical consensus of three plastic surgeons

**Table 2:** Full, verbatim structured AI prompt applied to Gemini 3 Flash.

**Full prompt applied to Gemini 3 Flash (verbatim; also provided as Supplementary File S1):**	
**Role:**	You are a specialized AI system designed for postoperative microvascular flap monitoring. Your goal is to provide a quantitative and qualitative analysis of skin flap viability based on the provided smartphone video.
**Analysis Protocol:**	**1. Baseline Assessment:** Identify the flap’s primary color (e.g., Pale, Pink, Dusky, Cyanotic) and estimate the RGB/Hex values of the flap surface compared to the surrounding healthy skin.
	**2. Turgor & Surface Analysis:** Evaluate visual signs of edema, skin tension, or presence of surgical blebs/blisters.
	**3. Temporal CRT Analysis:** Detect the exact timestamp (s:ms) when the compression (blanching) is released. Detect the exact timestamp when the tissue returns to its pre-compression color. Calculate the absolute Capillary Refill Time (CRT) in milliseconds.
	**4. Vascular Classification:** Categorize the flap status based on the following criteria: Normal: Pink color, CRT 1–2 s. Arterial Insufficiency: Pale/White color, CRT > 2 s or absent. Venous Congestion: Blue/Purple color, CRT < 1 s (early) or CRT > 2 s/Sluggish (late stage).
	**5. Confidence Score & Data Integrity:** Overall Confidence Score (0–100%): Provide a numerical value representing the reliability of the analysis. Justification: Briefly explain the score based on video quality, lighting stability, and motion artifacts. Reliability Threshold: If the score is below 70%, provide a warning: “Caution: Low reliability due to [Factor]. Clinical correlation is mandatory.”

**Table 3:** Video acquisition protocol specifications.

Parameter	Specification
Device	Smartphone
Illumination	Natural daylight
Duration	Minimum 10 s
Distance	25 ± 5 cm, perpendicular to flap surface
CRT maneuver	Surgical instrument compression applied and released during recording
Anonymization	No patient-identifying features in frame
AI session reset	New session initiated per video; prior session cleared before next upload

CRT: capillary refill time; AI: artificial intelligence.

**Table 4:** Agreement between AI and clinician assessments for flap colour.

AI Flap Colour	Reference: Pink (n = 96)	Reference: Blue/Purple (n = 43)	Reference: Pale/White (n = 4)	Total
Pink (n = 85)	78 (91.8%)	7 (8.2%)	0 (0.0%)	85
Blue/Purple (n = 44)	11 (25.0%)	33 (75.0%)	0 (0.0%)	44
Pale/White (n = 14)	7 (50.0%)	3 (21.4%)	4 (28.6%)	14
**Total**	**96**	**43**	**4**	**143**

Cohen’s kappa analysis. Data presented as n (row percentages). Overall agreement: κ = 0.613 (95% CI: 0.489–0.737); percent agreement = 80.4%. *p* < 0.001.

**Table 5:** Agreement between AI and clinician assessments for turgor.

AI Turgor	High (n = 54)	Low (n = 2)	Normal (n = 87)	Kappa (95% CI)	Compliance
High (n = 62)	47 (75.8%)	2 (3.2%)	13 (21.0%)	0.676 (0.558–0.794)	83.9%
Low (n = 1)	0 (0.0%)	0 (0.0%)	1 (100.0%)		
Normal (n = 80)	7 (8.8%)	0 (0.0%)	73 (91.3%)		

Cohen’s kappa analysis. Data presented as n (row percentages). *p* < 0.001 for all.

**Table 6:** Agreement between AI and clinician assessments for capillary refill time.

AI CRT	Brisk (n = 44)	Normal (n = 90)	Prolonged (n = 9)	Kappa (95% CI)	Compliance
Brisk (n = 38)	27 (71.1%)	8 (21.1%)	3 (7.9%)	0.487 (0.360–0.614)	74.1%
Normal (n = 86)	13 (15.1%)	72 (83.7%)	1 (1.2%)		
Prolonged (n = 19)	4 (21.1%)	10 (52.6%)	5 (26.3%)		

Cohen’s kappa analysis. Data presented as n (row percentages). *p* < 0.001 for all. CRT: capillary refill time.

**Table 7:** Descriptive statistics for AI-calculated capillary refill time and confidence score.

Variable	Mean ± SD	Median (Range)
AI-calculated CRT (s)	1.59 ± 1.25	1.4 (0.3–9.4)
Confidence score	87.54 ± 5.42	88 (55–95)

SD: standard deviation; CRT: capillary refill time.

**Table 8:** Agreement between AI and clinician assessments for clinical diagnosis.

Real Diagnosis (Expert Consensus)/AI Diagnosis	Normal (n = 82)	Venous Compromise (n = 47)	Arterial Insuff. (n = 14)	Kappa (95% CI)	Compliance
Normal (n = 84)	68 (81.0%)	9 (10.7%)	7 (8.3%)	0.524 (0.399–0.649)	74.1%
Venous Compromise (n = 49)	12 (24.5%)	34 (69.4%)	3 (6.1%)		
Arterial Insuff. (n = 10)	2 (20.0%)	4 (40.0%)	4 (40.0%)		

Rows represent the expert clinical consensus (reference); columns represent the AI classification. Percentages are row-wise (i.e., per-class sensitivity/recall relative to the expert clinical consensus). Cohen’s kappa analysis. *p* < 0.001 for all.

**Table 9:** Per-class classification metrics for clinical diagnosis, relative to expert clinical consensus.

Class	n	Sensitivity (95% CI)	Specificity (95% CI)	PPV (95% CI)	NPV (95% CI)	F1-Score
Normal	84	81.0% (70.9–88.7%)	76.3% (63.4–86.4%)	82.9% (73.0–90.3%)	73.8% (60.9–84.2%)	0.819
Venous Compromise	49	69.4% (54.6–81.7%)	86.2% (77.5–92.4%)	72.3% (57.4–84.4%)	84.4% (75.5–91.0%)	0.708
Arterial Insufficiency *	10	40.0% (12.2–73.8%)	92.5% (86.6–96.3%)	28.6% (8.4–58.1%)	95.3% (90.2–98.3%)	0.333
Overall accuracy	143	74.1%	—	—	—	—

PPV: positive predictive value; NPV: negative predictive value. Sensitivity/specificity denominators are given in the n column; PPV and NPV denominators (total AI-predicted positive/negative for each class, respectively) differ and are: Normal, n = 82/61; Venous Compromise, n = 47/96; Arterial Insufficiency, n = 14/129. F1-scores are reported as point estimates only (see Section 2.6). * Arterial insufficiency estimates are based on n = 10 and should be interpreted as exploratory given the wide confidence intervals.

**Table 10:** Binary classification metrics: compromised (venous compromise + arterial insufficiency) versus normal flap.

Comparison	n (Compromised/Normal)	Sensitivity (95% CI)	Specificity (95% CI)	PPV (95% CI)	NPV (95% CI)	F1-Score/Accuracy
Compromised vs. Normal	59/84	76.3% (63.4–86.4%)	81.0% (70.9–88.7%)	73.8% (60.9–84.2%)	82.9% (73.0–90.3%)	F1 = 0.750; Acc. = 79.0%

PPV: positive predictive value; NPV: negative predictive value. PPV denominator (total AI-predicted compromised) n = 61; NPV denominator (total AI-predicted normal) n = 82.

**Table 11:** Classification metrics of the confidence-score-based classifier (cutoff = 89) for pathological versus normal target classes.

Target Class	n	Sensitivity (95% CI)	Specificity (95% CI)	PPV (95% CI)	NPV (95% CI)	F1-Score
Pathological	59	77.0% (64.5–86.8%)	52.4% (41.1–63.6%)	54.7% (43.5–65.4%)	75.4% (62.2–85.8%)	0.644
Normal	84	52.4% (41.1–63.6%)	77.0% (64.5–86.8%)	75.4% (62.2–85.8%)	54.7% (43.5–65.4%)	0.618
**Overall accuracy**	**143**	**62.9% (54.5–70.9%)**	—	—	—	—

PPV: positive predictive value; NPV: negative predictive value. Confidence-score-based classification using a cutoff of 89: values below 89 were classified as diagnostic for a pathological result, and values above 89 as diagnostic for a normal result. AUC = 0.667 (95% CI: 0.579–0.755), *p* = 0.001. F1-scores are reported as point estimates only (see Section 2.6).

## Data Availability

The raw data supporting the conclusions of this article will be made available by the authors on request.

## Supplementary Materials

The following supporting information can be downloaded at https://www.mdpi.com/article/10.3390/jcm15156114/s1: the full, verbatim structured prompt supplied to Gemini 3 Flash for video analysis (Supplementary File S1).
